# miR-486-5p suppresses autophagy in hepatocellular carcinoma via activation of the AKT/mTOR signalling pathway

**DOI:** 10.1186/s43556-026-00556-8

**Published:** 2026-08-24

**Authors:** Yazheng Li, Mei Hou, Yong Jin, Xiangjin Zhuang, Yueyi Li, Wen Yang, Hongyang Wang, Qiyu Feng

**Affiliations:** 1 Cancer Research Center, The First Affiliated Hospital of USTC, Division of Life Sciences and Medicine, University of Science and Technology of China, Hefei, Anhui 230001 China; 2 Eastern Hepatobiliary Surgery Institute/Hospital, International Cooperation Laboratory on Signal Transduction, Naval Medical University (Second Military Medical University), Shanghai, 200438 China; 3 National Center for Liver Cancer, Naval Medical University (Second Military Medical University), Shanghai, 201805 China; 4 Center for MPACC & MEM, Huazhong University of Science and Technology, Wuhan, 430074 China

**Keywords:** Autophagy, Hepatocellular carcinoma, MiR-486-5p, PTEN, MYB

## Abstract

Autophagy is controlled by a complex signalling network and is closely linked to the initiation and progression of hepatocellular carcinoma (HCC). However, how microRNAs (miRNAs) regulate autophagy in HCC remains incompletely understood. In this study, we identified microRNA-486-5p (miR-486-5p) as a tumour-associated microRNA that is enriched in circulating exosomes derived from patients with HCC. Elevated miR-486-5p promoted proliferation and suppressed apoptosis in HCC cell lines under stress conditions. Mechanistically, miR-486-5p directly targets the 3′ untranslated region of the phosphatase and tensin homologue (*PTEN*) gene, thereby decreasing the production of the PTEN protein and consequently leading to the activation of the protein kinase B (AKT)/mammalian target of rapamycin (mTOR) signalling pathway. This phenomenon was accompanied by the inhibitory phosphorylation of unc-51 like autophagy-activating kinase 1 (ULK1), which resulted in impaired autophagy. The pharmacological inhibition of AKT or mTOR led to the restoration of autophagy and the attenuation of miR-486-5p-driven growth and survival advantages. Furthermore, the transcription factor MYB was found to bind the promoter of *ankyrin-1* (*ANK1*), the host gene of miR-486-5p, and to drive its expression. In an orthotopic liver tumour model, the liver-targeted delivery of miR-486-5p accelerated tumour expansion, an effect that was reversed by AKT or mTOR blockade. Collectively, these findings delineate a MYB/miR-486-5p/PTEN/AKT/mTOR signalling axis that constrains autophagy to facilitate HCC progression, suggesting potential avenues for biomarker development and therapeutic intervention.

**Supplementary Information:**

The online version contains supplementary material available at 10.1186/s43556-026-00556-8.

## Introduction

Autophagy refers to an evolutionarily conserved catabolic process that sustains intracellular homeostasis through the lysosomal degradation and recycling of cytoplasmic components [1]. In cancer, autophagy exerts dual biological functions: it can either suppress or fuel tumour progression, depending on tumour subtype, disease stage, and surrounding microenvironmental stimuli [1, 2]. Autophagy can facilitate immune evasion in pancreatic ductal adenocarcinoma and promote metastasis in hepatocellular carcinoma (HCC) [3, 4], whereas its suppression in non-small cell lung cancer may promote metastasis [5]. This functional duality is particularly relevant in HCC, where defective hepatocytic autophagy can contribute to tumorigenesis, whereas aberrant activation of autophagy in tumour cells promotes epithelial–mesenchymal transition and metastatic progression [6, 7].

Autophagy regulates tumour development via multiple biological routes, including alleviating intracellular oxidative stress and maintaining genome stability [8, 9]. In certain contexts, excessive autophagy can induce autophagic cell death and limit tumour growth [10, 11]. Among the signalling cascades governing this process, the phosphoinositide 3-kinase (PI3K)/protein kinase B (AKT)/mammalian target of rapamycin (mTOR) axis serves as a central negative regulator of autophagy and a key driver of cell growth and survival [12–14]. In HCC, suppressor of cytokine signalling 5 (SOCS5) activates the mTOR pathway, thereby promoting metastasis [15]. Similarly, alpha-fetoprotein (AFP) inhibits autophagy through PI3K/AKT/mTOR signalling, enhancing proliferation and invasion [16]. The loss of nitrogen permease regulator-like 2 (NPRL2) has been shown to result in mTOR hyperactivation and suppression of autophagy [17]. Phosphatase and tensin homologue (PTEN), frequently downregulated in HCC tissues, negatively modulates PI3K/AKT signalling and is tightly linked to autophagy regulation [18, 19].

MicroRNAs (miRNAs) are critical post-transcriptional regulators of autophagy and have emerged as key modulators of this process in various cancers [20–22]. Given that autophagy is governed by an intricate signalling network whose dysregulation is intimately linked to HCC pathogenesis, probing how miRNAs intersect with this pathway offers a well-founded and productive entry point for identifying new regulatory mechanisms in liver cancer. These small non-coding RNAs typically bind to the 3′ UTR of target messenger RNAs, accelerating mRNA degradation or inhibiting translational efficiency, thereby modulating core cellular processes including proliferation, differentiation and cell survival [23, 24]. A number of miRNAs have been shown to modulate autophagy via diverse pathways in various cancer types. For instance, miR-106a-5p has been shown to suppress autophagy by targeting BTG3 in nasopharyngeal carcinoma [25]. miR-519a enhances autophagy by downregulating STAT3/Bcl-2 signalling in glioblastoma [26]. In breast cancer, miR-142-3p suppresses autophagic activity by activating AKT/mTOR signalling, thereby rendering cells more susceptible to chemotherapy [27]. However, the specific miRNA networks that link PTEN status to autophagic remodelling in HCC remain incompletely mapped.

In the present study, we identify microRNA-486-5p (hereafter referred to as “miR-486-5p”) as a microRNA whose expression is elevated in HCC and is transcriptionally driven by the oncogenic factor MYB. We show that miR-486-5p directly targets *PTEN* mRNA, relieving a critical brake on AKT/mTOR signalling and suppressing autophagy. The findings of the present study delineate a coherent regulatory circuit—MYB/miR-486-5p/PTEN/AKT/mTOR—that constrains autophagy, thereby facilitating HCC progression. These findings may offer opportunities for the development of biomarkers or targeted interventions.

## Results

### Identification and characterization of HCC-associated miRNAs

**Fig. 1:**
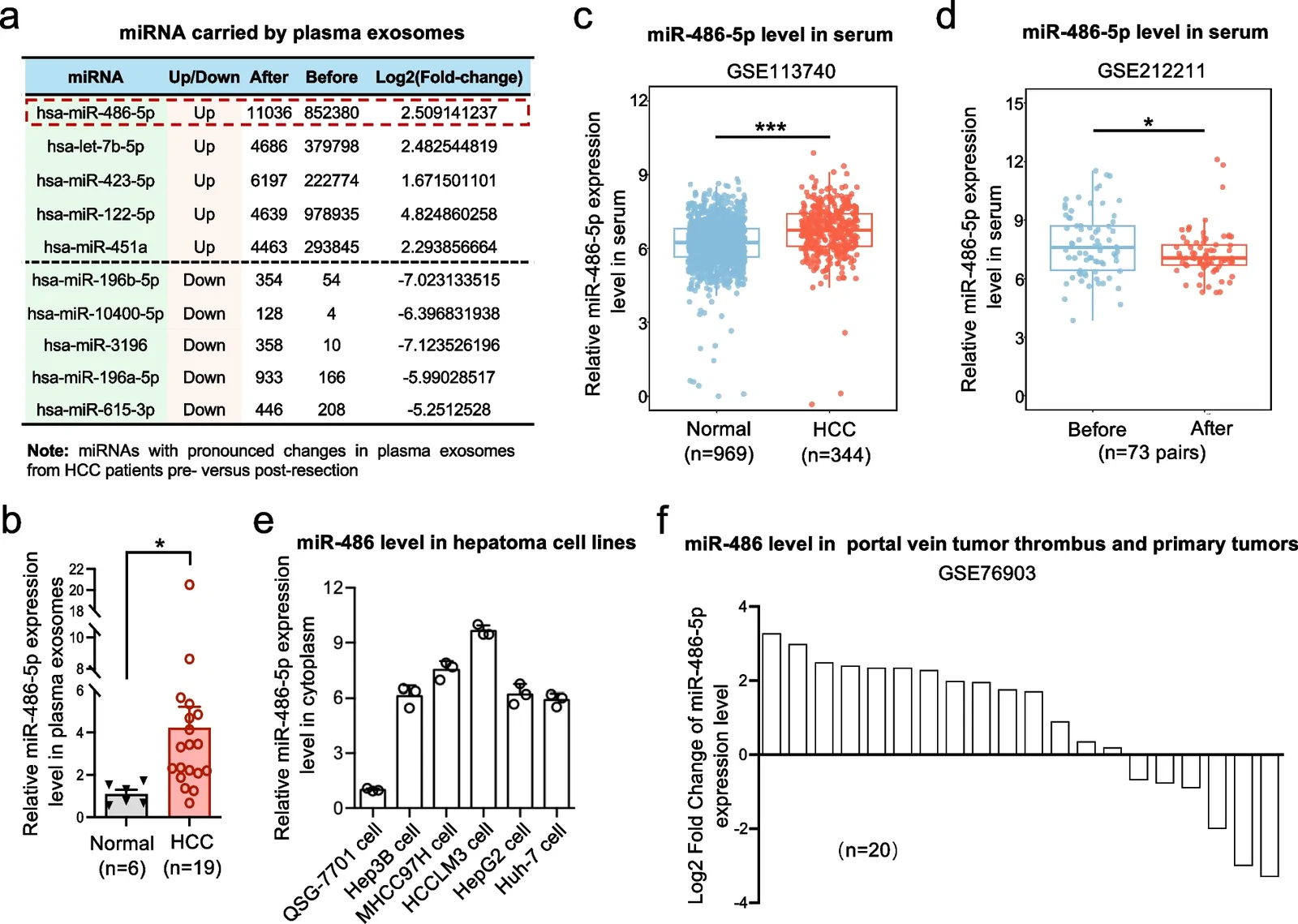
Identification and characterization of hepatocellular carcinoma (HCC)-associated microRNAs (miRNAs). **a** MiRNAs whose expression markedly changed in plasma exosomes from patients with HCC pre- versus postresection (*n* = 5 pairs). Log₂ fold change was calculated as log₂(Before/After) using the processed expression data.** b** Real-time quantitative PCR (RT‒qPCR) analysis of miR-486-5p levels in plasma exosomes from healthy donors (*n* = 6) and patients with HCC (*n* = 19). Mean ± standard error of the mean (SEM). **c** miR-486-5p expression in serum samples from normal controls (*n* = 969) and patients with HCC (*n* = 344) in the GSE113740 dataset. **d** miR-486-5p levels in patients with HCC pre- and postresection from GSE212211 (*n* = 73 pairs). **e** Relative miR-486-5p expression in HCC cell lines (Hep3B, MHCC97H, HCCLM3, HepG2 and Huh-7) versus normal hepatocytes (QSG-7701) (*n* = 3). Mean ± standard deviation (SD). **f** Log₂ fold change in miR-486-5p expression in portal vein tumour thrombi relative to matched primary tumours from the GSE76903 dataset (*n* = 20). (*p < 0.05; **p < 0.01; ***p < 0.001)

To systematically identify microRNAs associated with hepatocellular carcinoma progression, small RNA profiles in plasma exosome samples collected from patients before and after curative tumour resection were examined. MicroRNAs are small non-coding transcripts that mediate post-transcriptional gene regulation and participate in fundamental biological processes such as cell proliferation, metabolic reprogramming and signal transduction [28, 29]. Accumulating evidence has confirmed widespread miRNA dysregulation in various human cancers, with miRNAs functioning as either oncogenic drivers or tumour suppressors according to their downstream target genes [30]. Among the differentially expressed miRNAs identified in our previous sequencing analysis of plasma exosomes from patients with HCC [31], miR-486-5p expression markedly decreased after surgery (Fig. 1a). Further analysis showed that plasma exosomal miR-486-5p levels were significantly elevated in patients with HCC relative to healthy controls (Fig. 1b).

To further characterize the circulating miR-486-5p levels, publicly available datasets from the Gene Expression Omnibus (GEO) database were analysed. Serum levels of miR-486-5p were markedly higher in patients with HCC than in noncancer controls in the GSE113740 dataset (Fig. 1c). Furthermore, a decrease in serum levels of miR-486-5p was observed following tumour resection in the GSE212211 dataset (Fig. 1d), thereby substantiating its potential as a circulating surrogate marker of tumour burden.

Basal expression analysis showed that miR-486-5p was significantly elevated across the tested HCC cell lines relative to the normal hepatocyte cell line (Fig. 1e). Furthermore, analysis of the GSE76903 dataset demonstrated higher levels of miR-486-5p in portal vein tumour thrombus tissues than in matched primary tumours in 14 of 20 matched sample pairs (Fig. 1f). Portal vein tumour thrombus, a hallmark of advanced HCC, is associated with poor prognosis [32]. This enrichment suggests a role for miR-486-5p in malignant progression. Collectively, these findings identify miR-486-5p as a candidate HCC-associated microRNA with potential diagnostic and prognostic relevance.

### miR-486-5p regulates autophagy and apoptosis in hepatocellular carcinoma cells

**Fig. 2:**
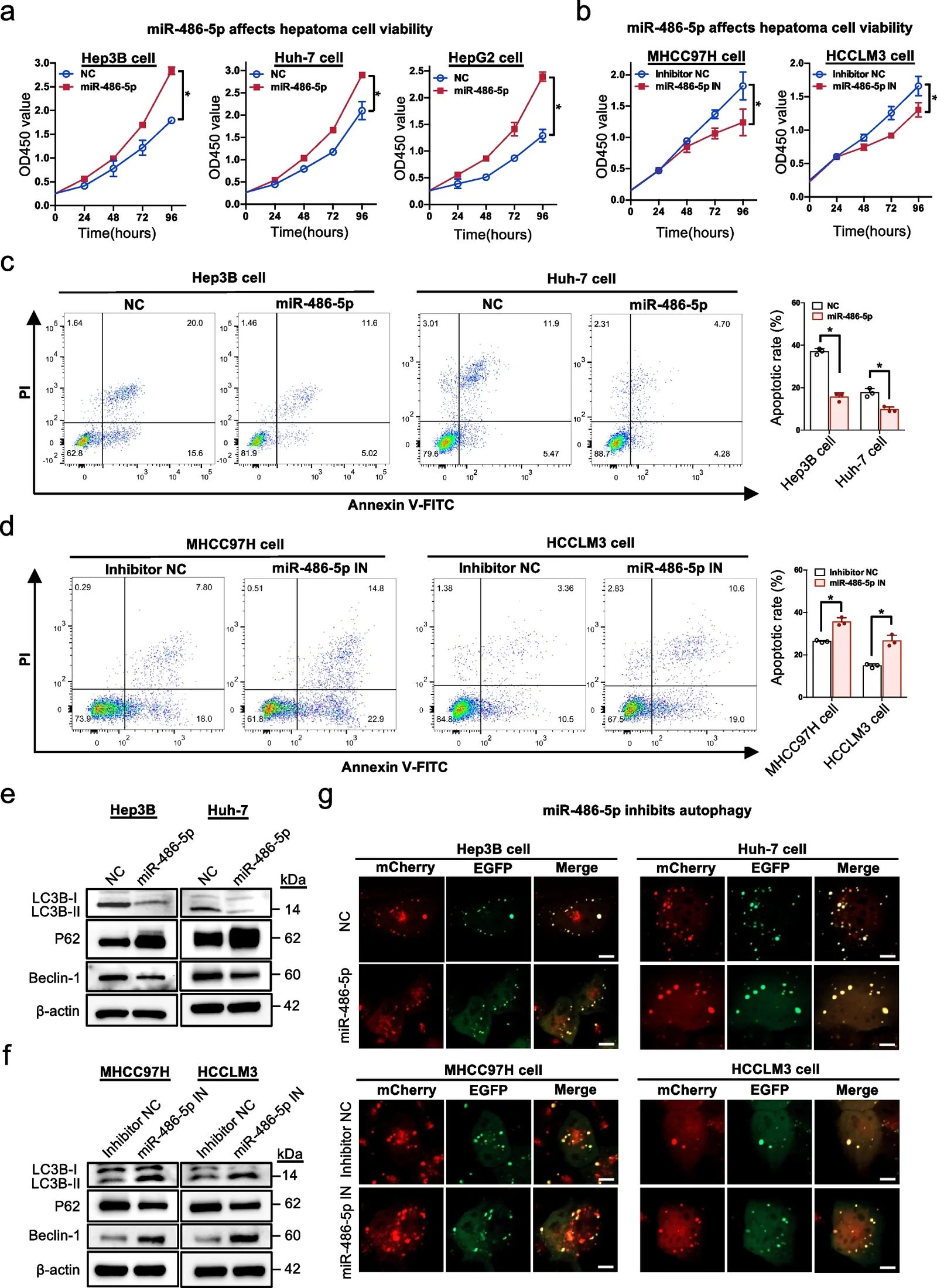
miR-486-5p regulates proliferation, apoptosis and autophagy in HCC cells. **a**, **b** Cell Counting Kit-8 (CCK-8) assays showing the viability of Hep3B, HepG2 and Huh-7 cells overexpressing miR-486-5p (**a**) and MHCC97H and HCCLM3 cells with miR-486-5p inhibition (**b**) (*n* = 3). **c**, **d** Flow cytometry analysis of apoptosis following miR-486-5p overexpression in Hep3B and Huh-7 cells (**c**) and inhibition in MHCC97H and HCCLM3 cells (**d**). The right panels show the quantification results (*n* = 3). **e**, **f** Western blot analysis of LC3B, p62 and Beclin-1 expression in Hep3B and Huh-7 cells overexpressing miR-486-5p (**e**) and in MHCC97H and HCCLM3 cells with miR-486-5p inhibition (**f**). **g** Confocal images of mCherry-EGFP-LC3-infected cells treated with control or miR-486-5p mimics in Hep3B and Huh-7 cells or inhibitors in MHCC97H and HCCLM3 cells. Green indicates EGFP-LC3, red indicates mCherry-LC3, yellow puncta indicate autophagosomes, and red-only puncta indicate autolysosomes. Scale bar, 10 μm. Mean ± SD. (*p < 0.05; **p < 0.01; ***p < 0.001)

To elucidate the functional significance of miR-486-5p in HCC, gain- and loss-of-function experiments were conducted under low-serum conditions that partially recapitulate the nutrient-limited tumour microenvironment. miR-486-5p overexpression was confirmed in HepG2, Huh-7 and Hep3B cells (Fig. S1a). Cell Counting Kit-8 (CCK-8) assays revealed that miR-486-5p overexpression promoted proliferation in these three lines (Fig. 2a), whereas its inhibition in MHCC97H and HCCLM3 cells curtailed growth (Fig. 2b). Considering the reciprocal balance between proliferation and apoptosis during tumour progression, we next assessed the influence of miR-486-5p on apoptosis. Flow cytometry analysis revealed that miR-486-5p overexpression reduced apoptotic ratios in Hep3B, Huh-7 and HepG2 cells (Fig. 2c, Fig. S1b), whereas its inhibition resulted in increased apoptosis in MHCC97H and HCCLM3 cells (Fig. 2d).

Given that autophagy has context-dependent effects on proliferation and survival in HCC [2], we next investigated whether this catabolic process is involved in the phenotypes driven by miR-486-5p. Immunoblotting revealed that the expression of miR-486-5p was accompanied by decreases in Beclin-1 and microtubule-associated protein 1A/1B-light chain 3B-II (LC3B-II) levels and the accumulation of the cargo receptor p62, suggesting a reduction in autophagic activity (Fig. 2e, Fig. S1c). Conversely, miR-486-5p inhibition in MHCC97H and HCCLM3 cells resulted in elevated Beclin-1 and LC3B-II levels, accompanied by a decrease in p62 expression (Fig. 2f, Fig. S1d). Autophagic flux was further assessed using the mCherry–EGFP–LC3 reporter. In this dual-fluorescence system, yellow puncta (mCherry⁺/EGFP⁺) are indicative of autophagosomes, whereas red puncta (mCherry⁺/EGFP⁻) correspond to autolysosomes, reflecting lysosomal fusion and EGFP quenching in the acidic environment. The overexpression of miR-486-5p markedly reduced the numbers of both yellow and red puncta in Hep3B and Huh-7 cells (Fig. 2g, Fig. S1e), whereas the inhibition of miR-486-5p increased the numbers of these puncta in MHCC97H and HCCLM3 cells (Fig. 2g, Fig. S1e), indicating enhanced autophagic flux. These findings indicate that miR-486-5p promotes HCC cell growth and survival, possibly through autophagy suppression.

### High *MYB* expression is associated with *ankyrin-1 (ANK1)* expression and poor prognosis in patients with HCC

**Fig. 3:**
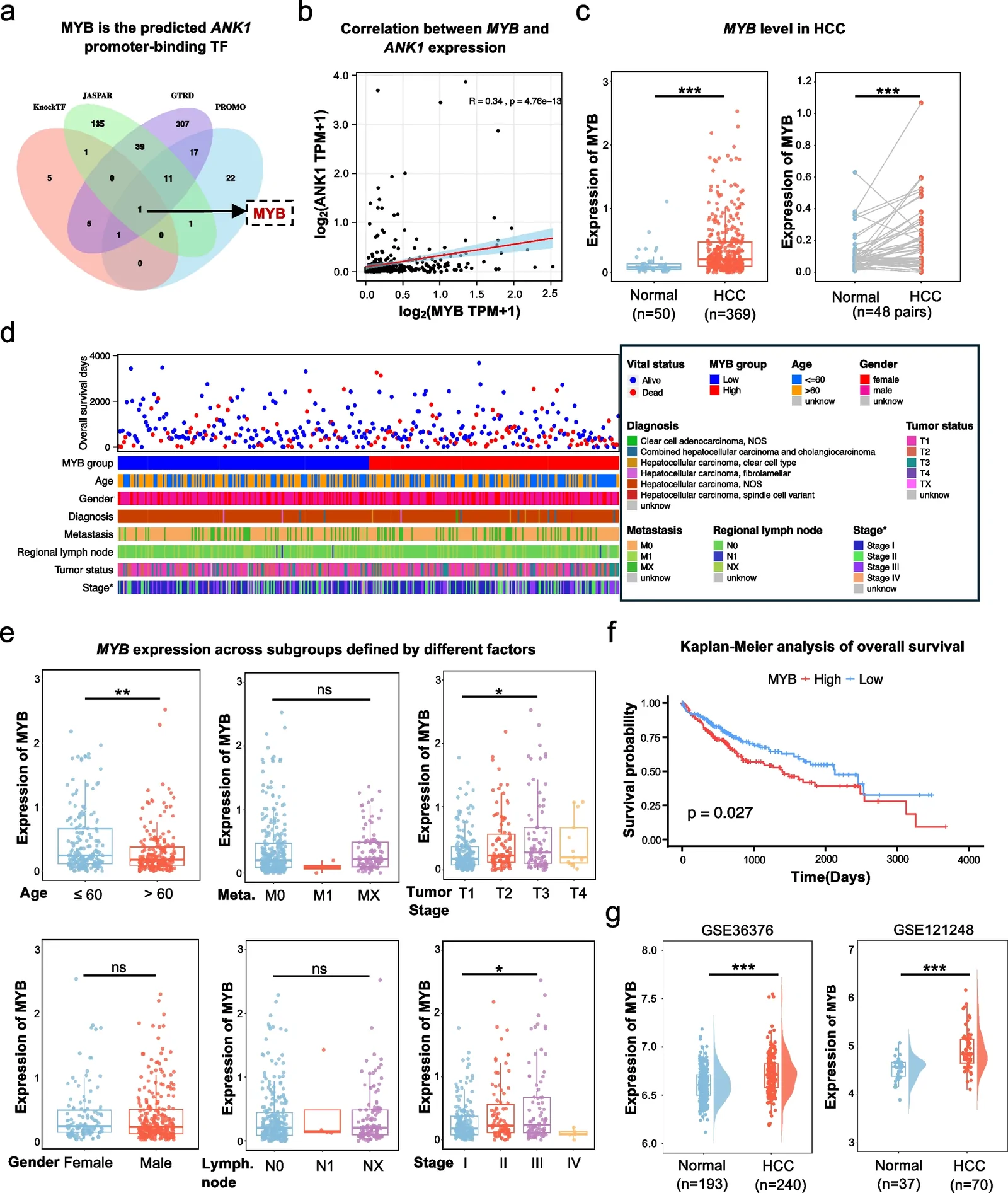
High *MYB* expression is associated with *ankyrin-1* (*ANK1*) expression and poor prognosis in patients with HCC. **a** Venn diagram showing MYB as the only predicted *ANK1* promoter-binding transcription factor (TF) from four databases. **b** Positive correlation between *MYB* and *ANK1* expression in The Cancer Genome Atlas Liver Hepatocellular Carcinoma (TCGA-LIHC) dataset. **c** *MYB* expression in normal liver tissues (*n* = 50) versus HCC tissues (*n* = 369) (left) and in paired HCC and adjacent nontumour tissues (*n* = 48 pairs) (right). **d** Clinicopathological profiles of patients with HCC stratified by *MYB* expression. **e** MYB expression across subgroups defined by different clinicopathological characteristics*.* **f** Kaplan–Meier analysis of overall survival in patients with HCC in the TCGA dataset stratified by *MYB* expression (*n* = 365). **g** *MYB* expression in tumour versus normal liver tissue from the GSE36376 (normal, *n* = 193; HCC, *n* = 240) and GSE121248 (normal, *n* = 37; HCC, *n* = 70) datasets. (*p < 0.05; **p < 0.01; ***p < 0.001; ns, not significant)

To further investigate the upstream regulatory mechanisms that may underlie the observed dysregulation of miR-486-5p, a comprehensive search was conducted for transcription factors that can regulate the promoter of *ANK1*, the host gene that harbours miR-486-5p. Transcription factors (TFs) are DNA-binding proteins that regulate transcriptional initiation and control expression of both protein-coding genes and non-coding RNAs including microRNAs [33–35]. It was hypothesized that miR-486-5p might be transcriptionally regulated in HCC, and further investigations were conducted to test this hypothesis. miR-486 is embedded within an intronic region of the *ANK1* gene and is known to be transcribed under the control of the *ANK1* promoter [36, 37]. By cross-referencing four databases (KnockTF, JASPAR, GTRD and PROMO), we identified MYB as the only overlapping candidate (Fig. 3a).

A subsequent analysis of The Cancer Genome Atlas Liver Hepatocellular Carcinoma (TCGA-LIHC) dataset revealed a positive correlation between *MYB* and *ANK1* expression (Fig. 3b). Subsequent analysis showed markedly higher MYB expression in HCC samples than in normal liver in both unpaired and paired comparisons (Fig. 3c). Patients with HCC who exhibited clinical and pathological characteristics were then stratified according to their *MYB* expression levels. Analysis of their clinicopathological features revealed elevated *MYB* expression in patients aged ≤ 60 years; however, no significant associations were observed with sex or nodal/metastatic stage, although higher *MYB* levels were noted in stage III tumours (Fig. 3d, e). Furthermore, *MYB* expression was strongly correlated with tumour status.

Kaplan‒Meier survival analysis of the TCGA-LIHC cohort (*n* = 365), stratified by median *MYB* expression, showed that patients with high *MYB* expression had shorter overall survival (Fig. 3f). Independent analyses of two GEO datasets (GSE36376 and GSE121248) further confirmed MYB upregulation in HCC tissues (Fig. 3g). These results suggest that MYB is a potential upstream regulator of miR-486-5p with prognostic relevance, prompting further functional studies.

### miR-486-5p is regulated by the transcription factor MYB

**Fig. 4:**
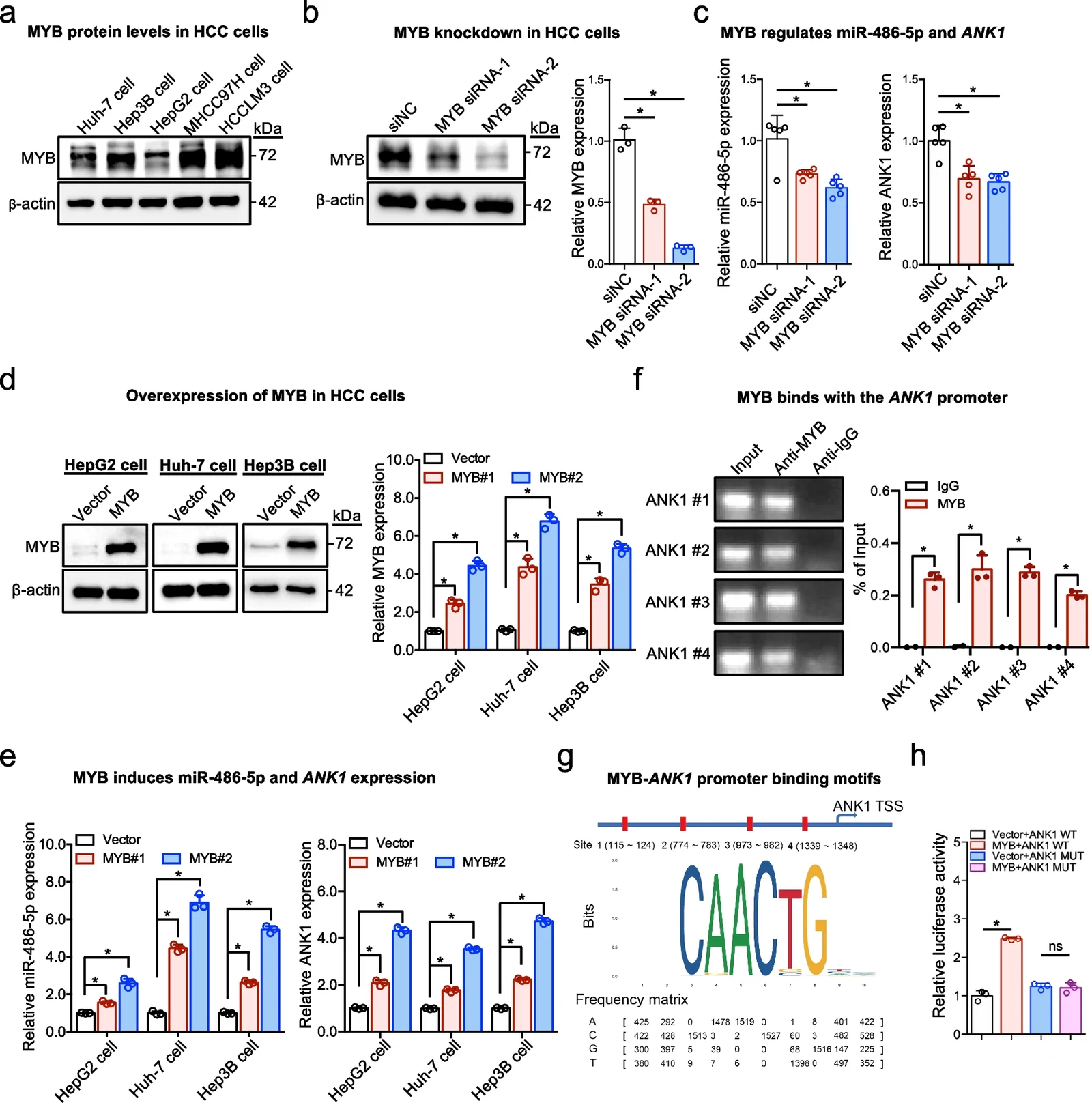
miR-486-5p is regulated by the transcription factor MYB. **a** Western blot showing MYB protein levels in HCC cell lines. **b** Western blot (left) and RT‒qPCR (right, *n* = 3) confirming MYB expression in HCCLM3 cells following MYB knockdown. **c** RT‒qPCR of miR-486-5p and ANK1 in MYB**-**knockdown HCCLM3 cells (*n* = 5). **d** Western blot (left) and RT‒qPCR (right, *n* = 3) results validating MYB overexpression in HepG2, Huh-7 and Hep3B cells following transfection with the MYB plasmid. **e** RT‒qPCR of miR-486-5p and ANK1 in MYB-overexpressing HepG2, Huh-7 and Hep3B cells (*n* = 3). **f** Chromatin immunoprecipitation (ChIP) assays showing MYB binding at four regions (ANK1#1-#4) within the *ANK*1 promoter. Left, gel electrophoresis; right, qPCR quantification (*n* = 3). **g** Schematic of MYB binding motifs upstream of the ANK1 transcription start site. **h** Dual-luciferase assays showing MYB-dependent activation of the *ANK1* promoter; mutation of binding motifs abolishes this effect (*n* = 3). Mean ± SD. (*p < 0.05; **p < 0.01; ***p < 0.001; ns, not significant)

To determine whether MYB regulates miR-486-5p, MYB protein levels were examined across HCC cell lines by western blotting. MYB levels were high in MHCC97H and HCCLM3 cells, moderate in Hep3B cells, and low in Huh-7 and HepG2 cells (Fig. 4a). MYB expression was knocked down in HCCLM3 cells using two independent siRNAs, as confirmed by western blotting and RT–qPCR (Fig. 4b). MYB knockdown significantly reduced the expression levels of both miR-486-5p and its host gene *ANK1* (Fig. 4c). Conversely, MYB was successfully overexpressed in HepG2, Huh-7 and Hep3B cells by transfection with a MYB expression plasmid, as confirmed by western blotting and RT–qPCR (Fig. 4d). RT–qPCR further revealed dose-dependent increases in the expression levels of both miR-486-5p and *ANK1* upon MYB overexpression (Fig. 4e). Potential MYB binding sites within the promoter region of ANK1, the host gene of miR-486-5p, were subsequently predicted using the JASPAR database. Chromatin immunoprecipitation (ChIP) assays were subsequently performed with primers targeting the predicted regions. The results demonstrated that MYB directly binds to the ANK1 promoter, which contains four MYB binding motifs (Fig. 4f, g). A dual-luciferase reporter assay revealed that MYB enhanced luciferase activity driven by the wild-type ANK1 promoter, whereas mutation of the predicted sites abolished this effect (Fig. 4h). These findings demonstrate that MYB directly binds to the ANK1 promoter and transcriptionally activates miR-486-5p, identifying a MYB-miR-486-5p axis that may contribute to HCC progression.

### miR-486-5p activates the AKT/mTOR signalling pathway by targeting PTEN

**Fig. 5:**
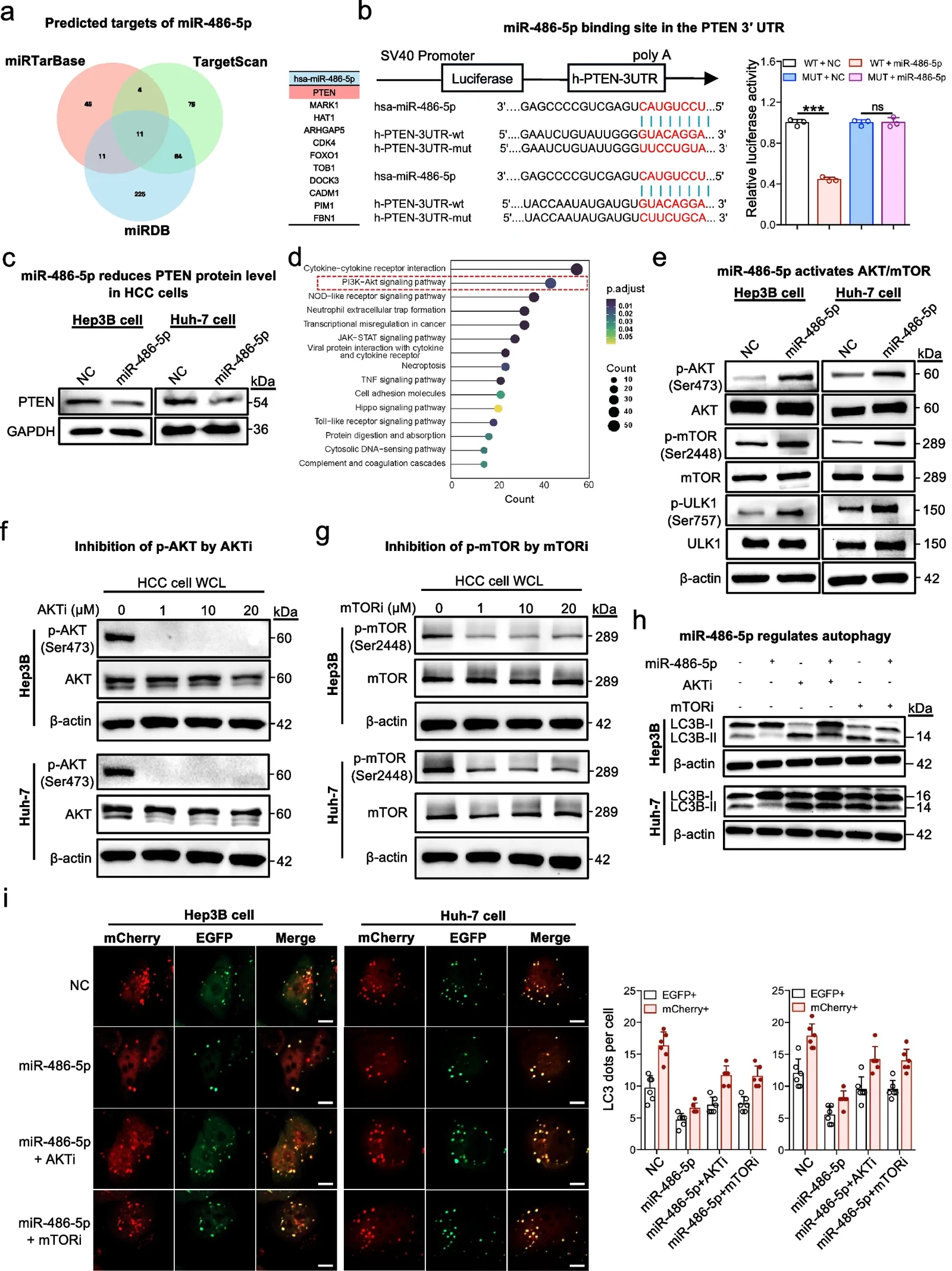
miR-486-5p activates the protein kinase B (AKT)/mammalian target of rapamycin (mTOR) signalling pathway by targeting phosphatase and tensin homologue (PTEN). **a** Predicted targets of miR-486-5p from TargetScan, miRTarBase and miRDB (left) and shared candidates (right), with PTEN highlighted. **b** Schematic representation of the binding sites of miR-486-5p in the 3′ untranslated region (UTR) of PTEN (left) and a luciferase reporter assay showing direct targeting of PTEN (right, *n* = 3). **c** Western blot showing decreases in the levels of PTEN protein in Hep3B and Huh-7 cells treated with miR-486-5p. **d** KEGG enrichment of differentially expressed genes (DEGs) in miR-486-5p-transfected hepatocytes (WRL68 cells). **e** Western blot of p-AKT (Ser473), AKT, p-mTOR (Ser2448), mTOR, p-ULK1 (Ser757) and ULK1 in Hep3B and Huh-7 cells treated with miR-486-5p or negative control. **f**, **g** Inhibition of p-AKT (**f**) and p-mTOR (**g**) by an AKT inhibitor (MK-2206, AKTi) and an mTOR inhibitor (rapamycin, mTORi), respectively. **h** Western blot showing LC3B levels in Hep3B and Huh-7 cells under the indicated treatments. **i** Confocal images of mCherry-EGFP-LC3-infected Hep3B and Huh-7 cells treated as indicated (left). Quantification of EGFP-positive and mCherry-positive LC3 puncta from the mCherry–EGFP–LC3 reporter images (right, *n* = 6). Green indicates EGFP-LC3, red indicates mCherry-LC3, yellow puncta indicate autophagosomes, and red-only puncta indicate autolysosomes. Scale bar, 10 μm. Mean ± SD. (*p < 0.05; **p < 0.01; ***p < 0.001; ns, not significant)

To elucidate the molecular mechanism of miR-486-5p in HCC, potential target genes were predicted using TargetScan, miRTarBase and miRDB. Eleven candidates were identified, among which *PTEN*, a well-characterized tumour suppressor, was of particular interest (Fig. 5a). Sequence alignment revealed two predicted miR-486-5p binding sites within the 3′ untranslated region (UTR) of PTEN. Luciferase reporters carrying the wild-type (WT) or mutant (MUT) 3′UTR of PTEN were generated to test direct targeting. Furthermore, cotransfection of cells with miR-486-5p and the WT reporter resulted in a substantial decrease in luciferase activity, whereas no such effect was observed with the MUT construct (Fig. 5b), confirming that PTEN was a direct target. Western blotting further showed that miR-486-5p overexpression significantly reduced PTEN protein levels in Hep3B and Huh-7 cells (Fig. 5c, Fig. S1f), thereby supporting PTEN as a direct target.

To investigate downstream signalling changes induced by miR-486-5p, we performed transcriptomic analysis of hepatocytes overexpressing miR-486-5p. KEGG pathway analysis identified significant enrichment of PI3K/AKT signalling in WRL68 cells overexpressing miR-486-5p (Fig. 5d), consistent with the known effects of PTEN loss on this signalling axis [38]. Consequently, the expression of miR-486-5p led to increases in the phosphorylation of AKT (Ser473), mTOR (Ser2448) and Unc-51 like autophagy activating kinase 1 (ULK1) (Ser757) (Fig. 5e, Fig. S1g). The latter is an inhibitory phosphorylation that dampens autophagy initiation. ULK1, a pivotal initiator of autophagy, serves as a critical link between miR-486-5p activity and autophagy suppression, thereby establishing a mechanistic relationship between these biological processes. Consistent with this model, the expression of MYB increased the phosphorylation of AKT (Ser473) in Hep3B cells (Fig. S1h), supporting a functional connection between MYB activity and AKT signalling.

Consequently, the involvement of the AKT/mTOR pathway in the suppression of autophagy by miR-486-5p was assessed using specific inhibitors. An AKT inhibitor (MK-2206) and an mTOR inhibitor (rapamycin) effectively reduced AKT and mTOR phosphorylation, respectively, in HCC cells across a range of concentrations (Fig. 5f, g). In Hep3B and Huh-7 cells overexpressing miR-486-5p, treatment with the AKT or mTOR inhibitor restored LC3B-II levels (Fig. 5h, Fig. S1i), suggesting the reactivation of autophagy. Autophagic flux reporter assays further demonstrated increased numbers of yellow puncta (autophagosomes, mCherry⁺/EGFP⁺) and red puncta (autolysosomes, mCherry⁺/EGFP⁻) following AKT or mTOR inhibition (Fig. 5i), reflecting enhanced autophagic flux. These results demonstrate that miR-486-5p suppresses autophagy by targeting PTEN and activating the AKT/mTOR signalling pathway.

### miR-486-5p promotes HCC progression by regulating autophagy

**Fig. 6:**
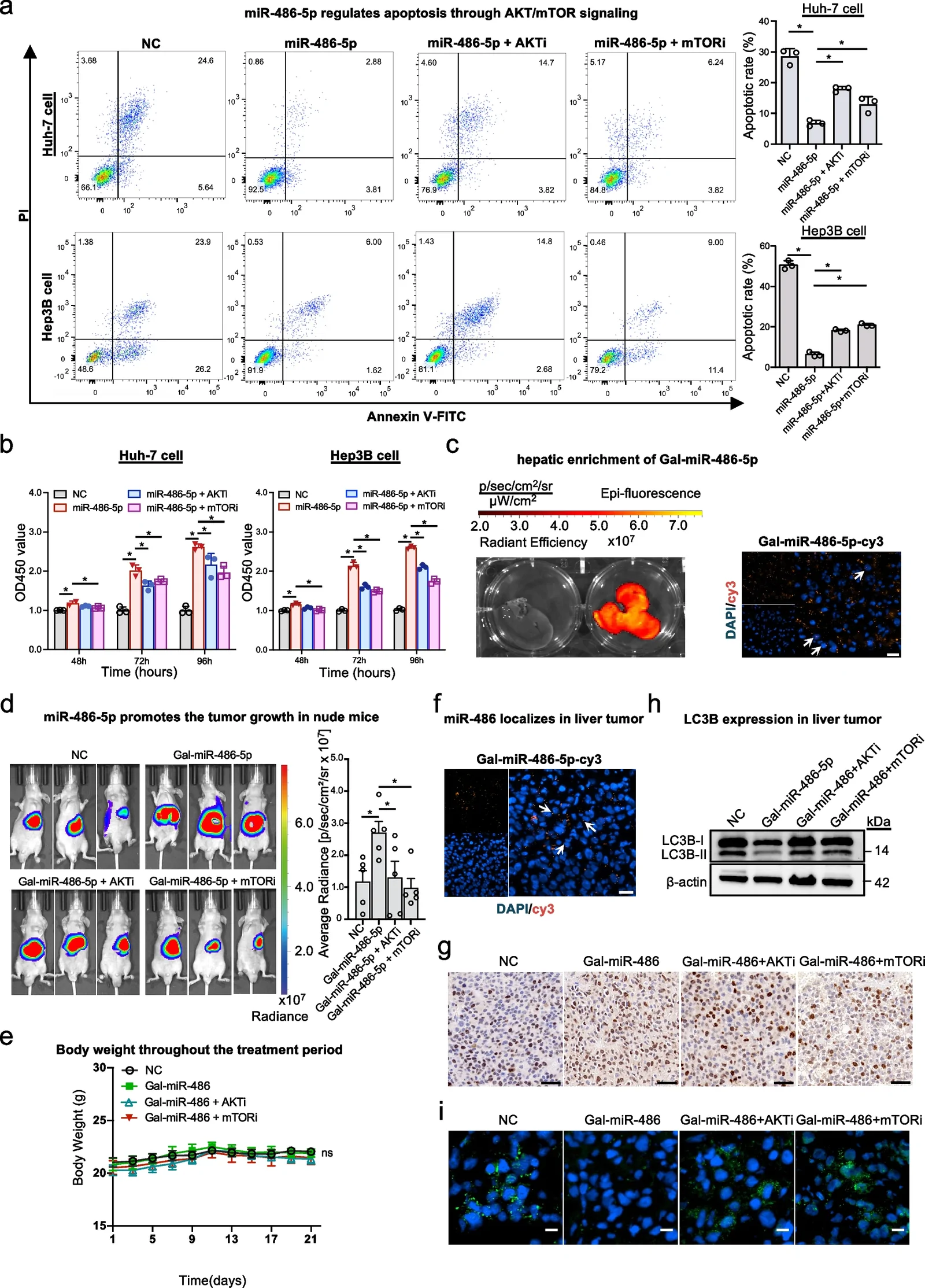
MiR-486-5p promotes HCC progression by regulating autophagy. **a** Flow cytometry analysis of apoptosis in Hep3B and Huh-7 cells following treatment with negative control (NC), miR-486-5p, miR-486-5p + AKT inhibitor (MK-2206), or miR-486-5p + mTOR inhibitor (rapamycin) (*n* = 3). **b** CCK-8 assays measuring the viability of Hep3B and Huh-7 cells at 48, 72 and 96 h after treatment with NC, miR-486-5p, miR-486-5p + AKT inhibitor, or miR-486-5p + mTOR inhibitor (*n* = 3). **c** Hepatic enrichment of Gal-miR-486-5p. Left, whole-organ epifluorescence imaging showing liver accumulation of Gal-miR-486-5p after tail vein injection. Right, representative liver sections showing Gal-miR-486-5p signals within liver tissue, with representative positive signals indicated by white arrows. Nuclei were counterstained with 4′,6-diamidino-2-phenylindole (DAPI, blue). Scale bar, 20 μm. **d** Bioluminescence imaging of orthotopic liver tumours in nude mice treated with negative control (NC), Gal-miR-486-5p, Gal-miR-486-5p + AKT inhibitor, or Gal-miR-486-5p + mTOR inhibitor. Representative images are shown. Average radiance was quantified (*n* = 5). Mean ± SEM. **e** Body weight measurements throughout the treatment period revealed no significant differences among groups (*n* = 5). **f** Fluorescence imaging showing Gal-miR-486-5p signals within liver tumour tissues, with representative positive signals indicated by white arrows. Nuclei were counterstained with DAPI (blue). Scale bar, 20 μm. **g** Immunohistochemical staining for Ki-67 in liver tumours from each group. Scale bar, 40 μm. **h** Western blot analysis of LC3B expression in liver tumours from the four groups. **i** Immunofluorescence staining of LC3B in liver tumours; DAPI was used for nuclear staining. Scale bar, 10 μm. The data are presented as the mean ± SD. (**a**, **b**). The data are presented as the mean ± SEM. (**d**, **e**). (*p < 0.05; **p < 0.01; ***p < 0.001; ns, not significant)

To determine whether pathway inhibition could neutralize the functional advantages conferred by miR-486-5p, apoptosis and proliferation were assessed following cotreatment with the inhibitors. The levels of apoptosis were assessed by flow cytometry, which revealed that the expression of miR-486-5p led to a significant reduction in apoptosis, whereas cotreatment with AKT or mTOR inhibitors restored the apoptosis rate (Fig. 6a, Fig. S2a). Concurrently, the results of the CCK-8 assays revealed increased cell proliferation in response to miR-486-5p overexpression, a phenomenon that was substantially suppressed by AKT or mTOR inhibition (Fig. 6b, Fig. S2b). These findings indicate that miR-486-5p promotes HCC cell survival and proliferation, at least in part through autophagy suppression.

To enable in vivo delivery, GalNAc-miR-486-5p-CY3 (Gal-miR-486-5p) was synthesized by labelling the 5′ end with Cy3 and conjugating the 3′ end with N-acetylgalactosamine, facilitating hepatocyte-specific targeting. After tail vein injection, pronounced fluorescence was detected in the liver, and immunofluorescence analysis revealed significant perinuclear accumulation of Gal-miR-486-5p in hepatocytes (Fig. 6c). In an orthotopic liver tumour model, bioluminescence imaging revealed that Gal-miR-486-5p increased tumour radiance, whereas cotreatment with either the AKT or mTOR inhibitor markedly reduced this signal (Fig. 6d). No significant differences in body weight were observed among the four groups throughout the study, indicating no overt treatment-related toxicity (Fig. 6e). Fluorescence imaging confirmed the substantial accumulation of Gal-miR-486-5p in tumour tissues, supporting its delivery to the tumour site and its potential to act on HCC cells (Fig. 6f). Consistent with the imaging results, Ki-67 immunohistochemistry revealed increased proliferative activity in tumours from Gal-miR-486-5p-treated mice, which was reduced upon AKT or mTOR inhibition (Fig. 6g, Fig. S2c). In Huh-7 subcutaneous xenografts, treatment with either the AKT or mTOR inhibitor alone did not significantly affect tumour volume or body weight compared with those of the vehicle control (Fig. S2e), excluding nonspecific antitumour effects.

**Fig. 7:**
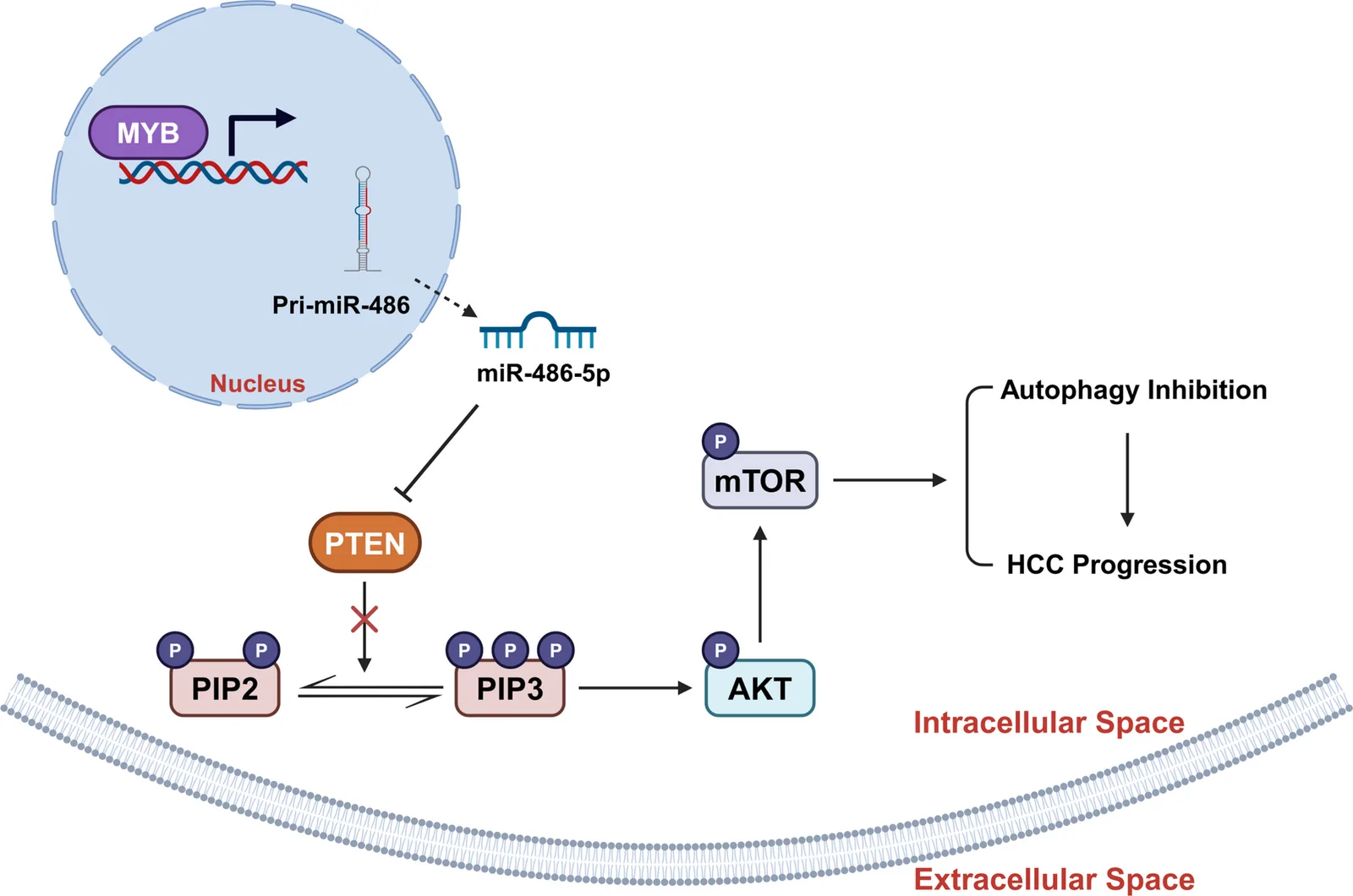
Schematic of miR-486-5p-mediated autophagy suppression in HCC. MYB activates miR-486-5p transcription via the ANK1 promoter. miR-486-5p represses PTEN expression, leading to the activation of AKT/mTOR signalling, autophagy suppression and tumour progression. Created with BioRender.com

The levels of autophagy were evaluated by assessing the expression of LC3B in tumour tissues. The results of both western blot and immunofluorescence analyses revealed that Gal-miR-486-5p reduced LC3B levels, indicating autophagy inhibition, whereas AKT or mTOR inhibition restored LC3B expression (Fig. 6h, i; Fig. S2d). Collectively, these results demonstrate that miR-486-5p promotes in vivo HCC progression by inhibiting autophagy in tumour cells. The oncogenic effect is significantly mitigated by blocking the AKT/mTOR pathway, suggesting that targeting miR-486-5p-driven malignancy is a potential therapeutic strategy (Fig. 7).

## Discussion

Liver cancer ranks sixth among globally diagnosed malignancies and third in cancer-associated mortality worldwide, imposing a severe public health burden across all continents [39]. HCC is characterized by extensive inter- and intratumoral heterogeneity, driven by subclonal diversity and multicentric tumour development within the same liver [40–42]. This inherent complexity is associated with a variety of pathological mechanisms and therapeutic resistance. High-throughput transcriptome sequencing advances have uncovered numerous dysregulated microRNAs exerting oncogenic or tumour-suppressive functions in HCC. Therefore, a more comprehensive understanding of the molecular networks that support HCC growth is essential to inform both prognostic assessment and therapeutic development. In the present study, we delineated a signalling cascade in which the transcription factor MYB drives the expression of miR‑486‑5p, which in turn reduces PTEN protein expression and unleashes AKT/mTOR signalling to suppress autophagy and promote tumour expansion.

The biological function of miR-486-5p appears highly context-dependent across distinct tumour models. Several prior studies reported reduced miR-486-5p abundance in HCC tissues and proposed tumour-suppressive roles for this miRNA [43, 44]. However, our data from patient exosomes, serum and a panel of HCC cell lines consistently showed elevated miR-486-5p levels. The expression of miR-486-5p varies depending on the cellular subtype, disease stage, or microenvironmental context. This variability is likely indicative of the heterogeneity of HCC. The decrease in circulating miR‑486‑5p levels following tumour resection further suggests that its extracellular abundance may serve as a surrogate indicator of disease burden. Notably, miR-486-5p is actively exported within exosomes; consequently, its intracellular abundance may not fully capture its biological impact, and interpretations based solely on bulk tissue expression should be made with caution. Emerging technologies, including single-cell and spatial transcriptomic approaches, provide methodological frameworks for resolving tumour cell states, microenvironmental context and spatial heterogeneity, which may help clarify the context-dependent behaviour of miR-486-5p in future studies [45].

In the present study, we identified the tumour suppressor PTEN as a direct target of miR-486-5p, thereby providing a molecular basis for its oncogenic function in HCC. Reduced PTEN expression is common in HCC and is associated with clinical outcome [18]. This classic tumour suppressor negatively controls PI3K/AKT cascade activity, a central signalling cascade that controls cell proliferation, metabolism and survival [46–48]. The loss or mutation of *PTEN* results in sustained activation of this pathway, thereby promoting tumour progression. By decreasing the protein abundance of PTEN, miR‑486‑5p relieves the inhibition of this axis, culminating in the phosphorylation of ULK1 at Ser757 and impaired autophagy initiation. The observation that pharmacological blockade of AKT or mTOR activity restored autophagy and attenuated the growth and survival advantages conferred by miR‑486‑5p underscores the centrality of this pathway to its oncogenic effects. These findings are consistent with a substantial body of research linking AKT/mTOR signalling to autophagy suppression [1, 49].

MYB is a key transcription factor that promotes proliferation, metastasis and immune evasion across diverse malignancies [50–52]. *MYB* was upregulated in HCC, and high MYB expression was associated with poor prognosis. Our study demonstrated that MYB directly binds to the promoter of *ANK1*, the host gene of miR-486-5p, and activates its transcription. These findings underscore a regulatory axis involving MYB and miR-486-5p, suggesting that MYB-driven transcriptional activation contributes to the aberrant expression of miR-486-5p in HCC.

From a translational and clinical perspective, the high abundance of miR-486-5p in patient plasma exosomes and its postoperative reduction support its potential as a minimally invasive biomarker for disease monitoring. Furthermore, tumours with elevated levels of miR-486-5p may rely strongly on AKT/mTOR signalling, suggesting that these tumours could be selectively vulnerable to pathway-targeted therapeutic interventions. Subsequent studies that examine the relationship between miR‑486‑5p levels and the response to AKT or mTOR inhibitors in preclinical models and patient cohorts will provide valuable insights into this possibility.

It is imperative to acknowledge the limitations of the present work. First, inhibitor-only treatments in an independent subcutaneous tumour model did not significantly affect tumour growth, making a major nonspecific antitumour effect less likely; however, matched controls were not included in the orthotopic experiment, and model-specific effects cannot be fully excluded. Second, although the key findings were validated across multiple HCC cell lines, the debated origin of HepG2 cells represents a limitation of this study and warrants caution in interpreting results derived from this cell line. Third, the clinical associations presented here require validation in larger, independent cohorts with linked treatment and outcome data. Fourth, although PTEN was identified as a direct target, the potential contribution of additional miR-486-5p effectors to the observed phenotypes warrants further investigation. The precise contribution of autophagy suppression relative to those of other AKT/mTOR-driven processes in mediating the oncogenic effects of miR-486-5p remains unclear. Future studies addressing these limitations will help refine the mechanistic framework proposed here. In summary, this study identifies a novel oncogenic pathway in HCC in which miR-486-5p directly targets PTEN to activate AKT/mTOR signalling and inhibit autophagy, thereby promoting tumour cell proliferation and survival. These findings underscore the therapeutic potential of targeting the miR-486-5p/PTEN/AKT/mTOR axis to inhibit HCC progression.

## Methods

### Reagents and antibodies

Primary antibodies against MYB (ab45150), Ki-67 (ab15580) and p62 (ab56416) were obtained from Abcam (Cambridge, UK). Anti-PTEN (A19104) was supplied by ABclonal (Wuhan, China), and anti-LC3B (NB100-2220) was obtained from Bio-Techne (Minnesota, USA). Antibodies against phospho-AKT (Ser473, 31957S), AKT (9272S), phospho-mTOR (Ser2448, 5536S), mTOR (2983S), phospho-ULK1 (Ser757, 6888T), ULK1 (8054T) and Beclin-1 (3495T) were acquired from Cell Signaling Technology (Danvers, MA, USA). Housekeeping protein antibodies including anti-GAPDH (60004–1-Ig) and anti-β-actin (66009–1-Ig), alongside horseradish peroxidase (HRP)-conjugated goat anti-rabbit and goat anti-mouse secondary IgG antibodies (SA00001-2, SA00001-1), were purchased from Proteintech (Wuhan, China). Alexa Fluor™ 488-labelled goat anti-rabbit IgG (H + L) (A-11008) was acquired from Invitrogen (Carlsbad, CA, USA).

The miR-486-5p mimics, inhibitors and mCherry-EGFP-LC3 adenovirus were obtained from HANBIO (Shanghai, China). GalNAc-modified miR-486-5p mimics were obtained from Hippo Biotechnology (Huzhou, China). MK-2206 (S1708) and rapamycin (HY-10219) were purchased from Selleck (Shanghai, China) and MedChemExpress (New Jersey, USA), respectively. The MYB-expressing plasmid (pcDNA3.1-MYB) was acquired from Beijing Tsingke Biotech Co., Ltd. (Beijing, China).

### Cell culture and transfection

MHCC97H, Huh-7, HCCLM3, WRL-68 and HEK293T cells were maintained in DMEM (VivaCell, Shanghai, China) supplemented with 10% foetal bovine serum (FBS; Lonsera, Uruguay). Hep3B and HepG2 cells were maintained in MEM (VivaCell, Shanghai, China), and QSG-7701 cells were maintained in RPMI-1640 (VivaCell, Shanghai, China), each with the same FBS proportion as mentioned before. The culture conditions for all cells are a humid environment with 5% CO₂ and a constant temperature of 37 °C. Short tandem repeat (STR) profiling was conducted to verify cell identity, and mycoplasma contamination detection was performed before all functional experiments.

When cell confluence reached 70%–80%, Lipofectamine 2000 transfection reagent (Invitrogen, Carlsbad, CA, USA) was applied for cell transfection following the manufacturer’s standard protocols. For MYB overexpression, 2.0 μg of MYB-expressing plasmid (pcDNA3.1-MYB) was used per well. Two independent siRNA sequences targeting MYB were utilized to establish MYB knockdown cell models. Plasmids and siRNA oligonucleotides that were diluted in Opti-MEM reduced serum medium (Gibco) were mixed with an equal volume of Opti-MEM containing Lipofectamine 2000. After incubation at room temperature for 20 min, the mixture was added dropwise to the cell monolayers. Unless otherwise specified, cells were collected 48 h post-transfection for subsequent molecular detection. The oligonucleotide primers and the designed sequences for MYB are listed in Supplementary Tables 1 and 2.

### Bioinformatics analysis

RNA-seq transcriptomic profiles, clinical metadata and survival data for HCC patient cohorts were downloaded from The Cancer Genome Atlas (TCGA) via the GDC official data portal (https://portal.gdc.cancer.gov). Publicly accessible gene expression microarray datasets GSE113740, GSE212211, GSE76903, GSE36376 and GSE121248 were retrieved from the NCBI Gene Expression Omnibus (GEO) database (https://www.ncbi.nlm.nih.gov/geo). All bioinformatic processing was executed using R statistical software (version 4.3.1). For transcription factor binding site prediction, the promoter region (2,000 bp upstream of the transcription start site) of the *ANK1* gene was analysed using JASPAR (v2024) and cross-referenced with the KnockTF (v2023), GTRD (v21.04) and PROMO (v3.0.2) databases. MYB was selected as the only overlapping transcription factor across all four databases. For miRNA target prediction, the 3′ UTR sequences of human genes were analysed using TargetScan, miRTarBase and miRDB. Eleven shared candidate target transcripts were identified, with PTEN prioritized for subsequent functional validation based on its well-established tumour-suppressive function in hepatic malignancies.

### Western blotting

Cells or tumour tissues were treated with RIPA lysis buffer (Beyotime Biotechnology, Shanghai, China) containing a fixed proportion of protease and phosphatase inhibitors (Beyotime Biotechnology, Shanghai, China). Post centrifugation at 12,000 rpm for a solid 15 min at a chilly 4 degrees Celsius, the resultant supernatant was carefully collected, and total concentrations were then quantified via the BCA assay (Thermo Fisher Scientific, Massachusetts, USA). Equal amounts of protein were subjected to SDS-PAGE and transferred onto 0.45 μm nitrocellulose membranes (Bio-Rad), blocked with a 5% solution of non-fat dry milk, diluted in TBST (Tris-buffered saline supplemented with 0.1% Tween-20) for 1 h at room temperature. Blots were incubated with primary antibodies overnight at 4 °C, followed by incubation with HRP-conjugated secondary antibodies (1:10,000 dilution) for 1 h at room temperature, and then visualized with SuperSignal West Pico PLUS chemiluminescent substrate (Thermo Fisher Scientific, Waltham, MA, USA). Band densitometry was quantified using ImageJ (v1.54, NIH), with target protein signals normalized to internal reference proteins GAPDH or β-actin.

### Quantitative real-time PCR (RT–qPCR)

Total cellular RNA was isolated through TRIzol reagent (Invitrogen, USA), and the concentration and purity were determined by a NanoDrop spectrophotometer (Thermo Fisher Scientific, Waltham, MA, USA). For mRNA detection, 1 μg total RNA was transcribed into complementary DNA utilizing the HiScript III 1st Strand cDNA Synthesis Kit (Vazyme Biotech, Nanjing, China) with oligo-dT primer templates. For miRNA detection, reverse transcription was completed by the Takara miRNA First-Strand Synthesis Kit (Takara, Kusatsu, Japan). Quantitative PCR amplification was carried out on a LightCycler 96 instrument (Roche, Basel, Switzerland) using AceQ SYBR Green qPCR Master Mix (Vazyme Biotech, Nanjing, China). The relative gene expression levels were calculated via the 2^−ΔΔCt^ method and normalized to those of *GAPDH* (coding transcript) or *U6* snRNA (microRNA transcript). All sequences are summarized in Supplementary Table 3.

### Chromatin Immunoprecipitation (ChIP)

ChIP experiments were carried out on HCCLM3 cells (4 × 10^6^ cells per condition) with a SimpleChIP Plus Enzymatic Chromatin IP Kit (Cell Signaling Technology, Danvers, MA, USA). Briefly, the cells were crosslinked with 1% formaldehyde for 10 min at room temperature, and then the reactions were terminated via addition of 125 mM glycine quenching solution. Cell pellets were resuspended in SDS lysis buffer supplemented with protease inhibitor cocktails, followed by chromatin fragmentation. Chromatin was sheared to fragments of 200–500 bp using a Bioruptor Pico sonicator (Diagenode; 12 cycles of 30 s on/30 s off at 4 °C). The fragmented chromatin solution was diluted tenfold with ChIP dilution buffer and incubated overnight at 4 °C with 5 μg of anti-MYB antibody (Abcam, ab45150) or normal rabbit IgG (negative control). Magnetic protein A/G bead complexes were added for 2 h at 4 °C and sequentially washed with low-salt, high-salt, lithium chloride-containing wash buffers, followed by two rounds of TE buffer rinsing. Immunoprecipitated DNA was eluted and reverse-crosslinked at 62 °C for 2 h, followed by proteinase K digestion. DNA was purified using spin columns and analysed by PCR and quantitative real-time PCR (qPCR) with primers flanking the predicted MYB-binding sites in the *ANK1* promoter. The primer sequences for the four MYB-binding regions (*ANK1*#1–#4) are provided in Supplementary Table 4.

### Luciferase reporter assays

For miRNA target validation, the wild-type human PTEN 3′ UTR containing the predicted hsa-miR-486-5p binding sites, reference transcript NM_000314.8, was synthesized and subcloned downstream of Renilla luciferase coding sequence within the psiCHECK-2 vector by HANBIO Biotechnology (Shanghai, China). The mutant reporter was generated by substituting the predicted seed-matching sequences. Specifically, the wild-type sequence GTACAGGA at positions 719–726 and 3186–3193 of the PTEN 3′ UTR was mutated to tTcCtGtA and cTtCtGcA, respectively.

For promoter activity assays, the human *ANK1* promoter region was cloned and inserted into the pGL3-Basic luciferase reporter vector. The mutant *ANK1* promoter reporter was generated by disrupting four putative MYB-binding motifs. The pRL-TK plasmid was used as an internal control for normalization in the *ANK1* promoter assays.

HEK293T cells seeded in 24-well plates were transfected using Lipofectamine 2000. For the PTEN 3′ UTR assay, cells were cotransfected with psiCHECK-2-PTEN-3′ UTR wild-type or mutant reporter plasmids together with miR-486-5p mimics or negative control mimics. For the *ANK1* promoter assay, cells were cotransfected with pGL3-*ANK1* promoter wild-type or mutant reporter plasmids, a MYB overexpression plasmid or an empty vector and pRL-TK. Forty-eight hours post-transfection, cellular luciferase activity was quantified using the Dual-Luciferase Reporter Assay System (Promega, Madison, WI, USA) following standard operating procedures.

### Cell viability assay

Cell viability was assessed using the Cell Counting Kit-8 (Biomiky, Shanghai, China). Cells (3,000/well) were seeded into 96-well plates and transfected as described above. At the designated intervals (0, 24, 48, 72 and 96 h), CCK-8 working solution was introduced into each well, followed by a two-hour incubation period. Following this, optical density readings were taken at a wavelength of 450 nm via a microplate reader (BioTek Synergy H1).

### Apoptosis assay

Apoptotic cell proportions were quantified using the application of an Annexin V-FITC/PI dual staining apoptosis detection kit (Beyotime Biotechnology, Shanghai, China). Following transfection, cells were collected and rinsed with pre-cooled phosphate-buffered saline (PBS) before being re-suspended in 500 µL of binding buffer. Next, 15 µL of annexin V-fluorescein isothiocyanate (FITC) and 5 µL of propidium iodide (PI) were introduced in sequence, and the mixture was left to incubate for 20 min, shielded from light. Fluorescence was then quantified within the hour on a flow cytometer (BD Biosciences, San Jose, CA, USA). Early apoptotic populations (annexin V⁺/PI⁻) and late apoptotic/necrotic populations (annexin V⁺/PI⁺) were combined to determine the total percentage of apoptotic cells. The data were analysed using FlowJo v10.8.

### Autophagic flux assay and confocal microscopy quantification

Cells were seeded at 1 × 10^4^ cells/dish in confocal culture dishes (Nest Biotechnology). After 24 h adherent culture, cells were infected with mCherry-EGFP-LC3 adenoviral particles at a multiplicity of infection (MOI) of 50 for 12 h, followed by culture medium replacement. Cells were transfected with miRNA mimics/inhibitors or treated with inhibitors as indicated. Forty-eight hours later, autophagic structures were visualized using a confocal microscope (Zeiss LSM 800, Jena, Germany) equipped with a 63 × oil immersion objective. mCherry was excited at 561 nm (emission 570–650 nm), and EGFP was excited at 488 nm (emission 500–550 nm). For each experimental condition, at least 15 randomly selected fields were imaged, and a minimum of 6 cells per group were analysed. Autophagic structures were manually counted by a blinded investigator using ImageJ. Yellow fluorescent puncta (mCherry⁺/EGFP⁺) were defined as autophagosomes, and red-only puncta (mCherry⁺/EGFP⁻) were defined as autolysosomes. Only puncta with a diameter > 0.2 μm and clearly distinguishable from the background were counted.

### Immunohistochemistry (IHC)

Tumour tissues were preserved in 4% paraformaldehyde, systematically dehydrated, embedded in paraffin, and sliced into 4-µm sections. The tissue sections underwent complete removal of paraffin, followed by rehydration, and then subjected to antigen retrieval using a pressure cooker with 10 mM citrate buffer (pH 6.0) for 2 min. To eliminate endogenous peroxidase activity, specimens were treated with 3% hydrogen peroxide for 10 min, prior to blocking nonspecific binding with 5% normal goat serum for one hour. The sections were then incubated overnight at 4 degrees Celsius with a primary anti-Ki-67 antibody (Abcam, ab15580; diluted 1:200), followed by application of a horseradish peroxidase-conjugated secondary antibody (1:500) for one hour at room temperature. Antigen–antibody complexes were visualized using a 3,3'-diaminobenzidine (DAB) chromogenic substrate (Vector Laboratories), while cell nuclei were counterstained with hematoxylin for contrast. For quantitative analysis, five randomly selected high-magnification fields per tumour section were captured using a light microscope (Nikon Eclipse Ci-L). The Ki-67 labeling index was determined by calculating the proportion of brown-stained nuclei relative to the total nuclear count.

### Immunofluorescence (IF)

To prepare frozen sections, OCT-embedded liver tumours were cryosectioned at a thickness of 5 μm, followed by permeabilization and blocking with 5% bovine serum albumin (BSA) diluted in PBS for 1 h at room temperature. Tissue slices were incubated with anti-LC3B primary antibody (1:200) overnight at 4 °C, then incubated with Alexa Fluor 488-labeled secondary antibody (1:500 dilution) for 1 h at room temperature. Cell nuclei were counterstained with 4′,6-diamidino-2-phenylindole (DAPI, 1 μg/mL). For tissue distribution observation of Cy3-labeled Gal-miR-486-5p, endogenous Cy3 red fluorescence was directly captured without additional antibody incubation. All immunofluorescence images were acquired via Zeiss Axio Imager.M2 fluorescence microscope with unified exposure parameters for all groups.

### Animal studies

Male BALB/c nude mice (Viton Lihua, Beijing, China), five weeks of age, were maintained under specific pathogen-free (SPF) conditions, exposed to a regular 12 h light/dark cycle, and provided with unrestricted access to both food and water.

For the orthotopic liver tumour model, luciferase-labelled Huh-7 cells (4 × 10⁶ cells) in 50 μL of PBS mixed with isopyknic Matrigel (Corning) were injected subcutaneously into the right axilla. After three weeks, visible tumours were excised, cut into ~1 mm^3^ fragments, and orthotopically implanted into the left liver lobe of recipient mice under isoflurane anaesthesia. Five days postimplantation, the mice were randomized into four groups (*n* = 5 per group) and treated via tail vein injection every 3 days for 3 weeks: (i) miRNA-NC (negative control, 40 nmol/kg); (ii) Gal-miR-486-5p (40 nmol/kg); (iii) Gal-miR-486-5p + AKT inhibitor (MK-2206, 2 mg/kg); and (iv) Gal-miR-486-5p + mTOR inhibitor (rapamycin, 2 mg/kg). The tumour burden was monitored weekly by bioluminescence imaging (IVIS Spectrum, PerkinElmer) after intraperitoneal injection of D-luciferin. Body weight was monitored every two days. At the study endpoint, the mice were euthanized by CO₂ inhalation followed by cervical dislocation, and liver tumours were harvested for further analysis.

For the subcutaneous xenograft model, Huh-7 cells (1 × 10⁶ cells suspended in 100 μL of PBS combined with Matrigel in equal volumes) were subcutaneously injected into the right axilla of each mouse to establish subcutaneous xenograft models. At 15 days post-inoculation, mice with established tumours were divided into three groups (*n* = 6 each) and received tail vein injections every 3 days with either vehicle (negative control), MK-2206 (2 mg/kg, AKT inhibitor), or rapamycin (2 mg/kg, mTOR inhibitor). The tumour volume (V) was measured every 4 days using a calliper and applying the calculation formula: V = (length × width^2^)/2. Body weight was monitored simultaneously to assess systemic toxicity. After 21 days of treatment, the mice were euthanized, and the subcutaneous tumours were dissected, photographed, and frozen or fixed for subsequent histological and molecular analyses.

### Statistical analysis

The data are reported as mean ± standard deviation (SD) for in vitro assays and mean ± standard error of the mean (SEM) for i*n vivo* studies, as specified in the corresponding figure captions. All statistical testing was carried out with GraphPad Prism version 9.5 (GraphPad Software, San Diego, CA, USA). To assess differences between two independent groups, unpaired two-tailed Student’s t-tests were employed; for matched or paired observations, paired two-tailed Student’s t-tests were used. When comparing three or more groups, one-way or two-way analysis of variance (ANOVA) was performed, with Tukey’s or Sidak’s post hoc corrections applied where suitable. Survival distributions were compared using the log-rank test in Kaplan–Meier analyses. Densitometric quantification of Western blots was conducted with ImageJ version 1.54. Statistical significance was defined as a p value below 0.05, with significance thresholds indicated as follows: *p < 0.05, **p < 0.01, ***p < 0.001, and ns (not significant).

## Supplementary Information

Supplementary Material 1: Supplementary Figure 1 Validation and quantification of the miR-486-5p-dependent effects on apoptosis, autophagy-associated markers and AKT/mTOR signalling. a. RT–qPCR analysis of miR-486-5p expression in HepG2, Huh-7 and Hep3B cells transfected with miR-486-5p mimics or the negative control (n = 3). b. Flow cytometry analysis and quantification of apoptosis in HepG2 cells transfected with miR-486-5p mimics or the negative control (n = 3). c. Quantification of microtubule-associated protein LC3B-II, p62 and Beclin-1 protein levels from the immunoblots shown in Fig. 2e (n = 3). d. Quantification of LC3B-II, p62 and Beclin-1 protein levels from the immunoblots shown in Fig. 2f (n = 3). e. Quantification of EGFP-positive and mCherry-positive LC3 puncta from the mCherry–EGFP–LC3 reporter images shown in Fig. 2 g (n = 6). f. Quantification of PTEN protein levels from the immunoblots shown in Fig. 5c (n = 3). g. Quantification of p-AKT/AKT, p-mTOR/mTOR and p-ULK1/ULK1 ratios from the immunoblots shown in Fig. 5e (n = 3). h. Immunoblot analysis and quantification of AKT phosphorylation in Hep3B cells following MYB overexpression (n = 3). i. Quantification of LC3B-II protein levels from the immunoblots shown in Fig. 5 h in cells treated with miR-486-5p mimics with or without AKT or mTOR inhibition (n = 3). Data are presented as the mean ± SD. (*p < 0.05; **p < 0.01; ***p < 0.001; ns, not significant). Supplementary Figure 2 Additional validation of AKT/mTOR-dependent rescue effects and *in vivo *controls. a. Flow cytometry analysis of apoptosis in HepG2 cells following treatment with negative control (NC), miR-486-5p, miR-486-5p+AKT inhibitor (MK-2206), or miR-486-5p+mTOR inhibitor (rapamycin) (n = 3). b. CCK-8 assays measuring the viability of HepG2 cells at 48, 72, and 96 h after treatment with NC, miR-486-5p, the miR-486-5p+AKT inhibitor, or the miR-486-5p+mTOR inhibitor (n = 3). c. Quantification of Ki-67-positive cells in liver tumour sections from mice treated with NC, Gal-miR-486-5p, Gal-miR-486-5p+AKT inhibitor or Gal-miR-486-5p+mTOR inhibitor (n = 5). d. Quantification of LC3B-II protein levels in liver tumour tissues from the indicated treatment groups (n = 3). e. Body weight, tumour volume and excised tumours from nude mice treated with NC, the AKT inhibitor or the mTOR inhibitor (n = 6). The data are presented as the mean ± SD. (a, b, c, d). The data are presented as the mean ± SEM. (e). (*p < 0.05; **p < 0.01; ***p < 0.001; ns, not significant).

## Data Availability

All the data generated or analysed during this study are included in this article and its supplementary information files. Public datasets analysed are available from the Gene Expression Omnibus under accessions GSE113740, GSE212211, GSE76903, GSE36376 and GSE121248. The TCGA-LIHC RNA-seq expression matrix and clinical information analysed in this study were downloaded from the official TCGA data platform, namely, the NCI Genomic Data Commons (GDC) Data Portal (https://portal.gdc.cancer.gov). The data used were all publicly accessible, deidentified open-access quantitative gene expression data and clinical information; no controlled-access TCGA data requiring dbGaP authorization were used.
