# Supplementary material for: miR-486-5p suppresses autophagy in hepatocellular carcinoma via activation of the AKT/mTOR signalling pathway
Source: Mol Biomed. 2026 Aug 24;7:148. doi: 10.1186/s43556-026-00556-8 (PMC13504038; doi:10.1186/s43556-026-00556-8)
Supplement: Supplementary file 1 [file 43556_2026_556_MOESM1_ESM.pdf]

miR-486-5p Suppresses Autophagy in Hepatocellular Carcinoma *via* Activation of the AKT/mTOR Signalling Pathway

Yazheng Li[1, #], Mei Hou[1, #], Yong Jin[1, 2, 3], Xiangjin Zhuang[1], Yueyi Li[4], Wen Yang[1, 2, 3, *], Hongyang Wang[1, 2, 3,*] and Qiyu Feng[1, 2, 3, *] (corresponding author)

[1]Cancer Research Center, The First Affiliated Hospital of USTC, Division of Life Sciences and Medicine, University of Science and Technology of China, Hefei 230001, Anhui, China

[2]Eastern Hepatobiliary Surgery Institute/Hospital, International Cooperation Laboratory on Signal Transduction, Naval Medical University (Second Military Medical University), Shanghai 200438, China

[3]National Center for Liver Cancer, Naval Medical University (Second Military Medical University), Shanghai 201805, China

[4]Center for MPACC & MEM, Huazhong University of Science and Technology, Wuhan 430074, China

[#] Contributed equally.

[*] Corresponding author.

Correspondence to Wen Yang (woodeasy66@hotmail.com), Hongyang Wang (hywangk@vip.sina.com), or Qiyu Feng (qiyu@icloud.com, qiyufeng@ustc.edu.cn)

Supplementary Figure 1 Validation and quantification of the miR-486-5p-dependent effects on apoptosis, autophagy-associated markers and AKT/mTOR signalling.

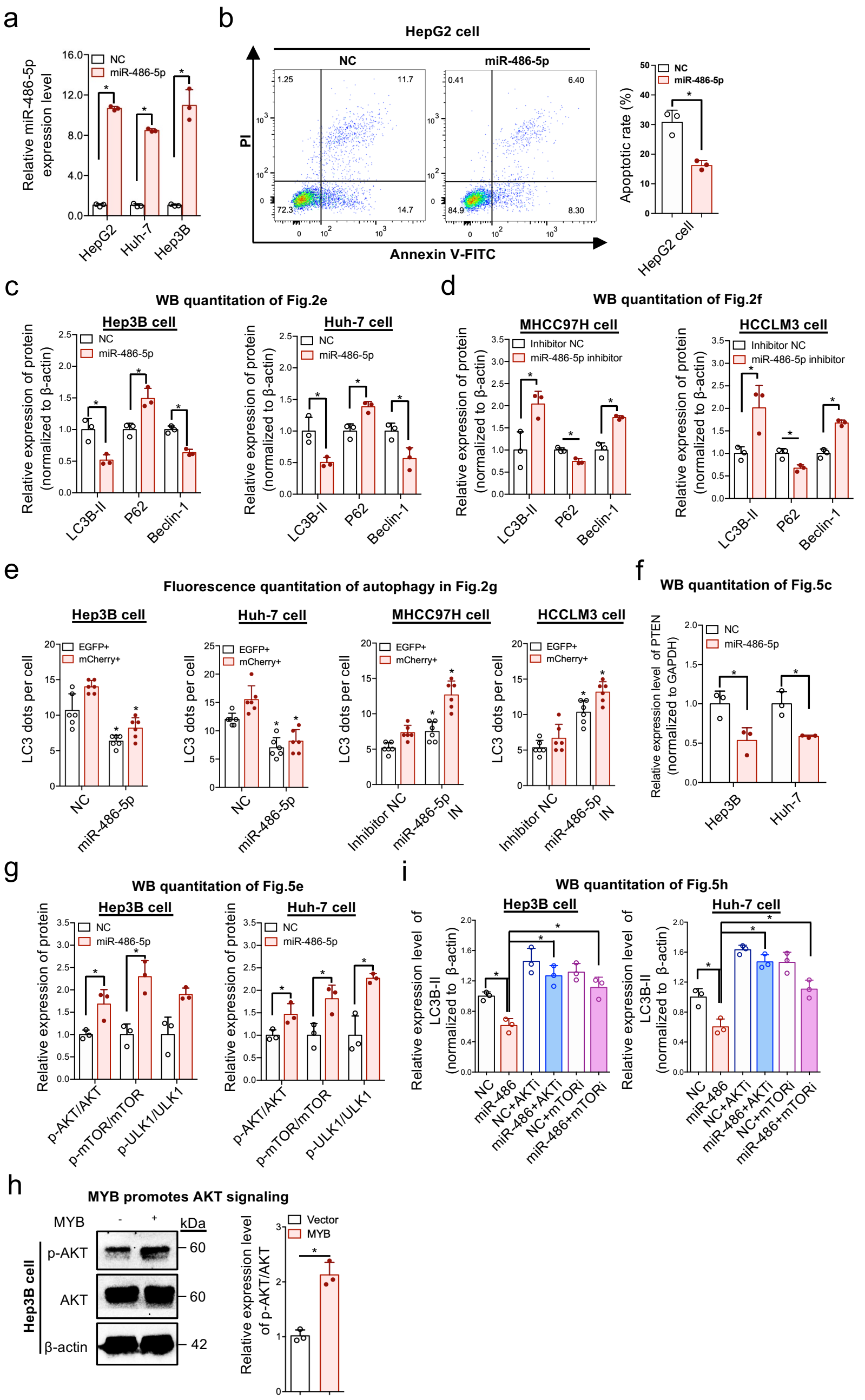

a. RT–qPCR analysis of miR-486-5p expression in HepG2, Huh-7 and Hep3B cells transfected with miR-486-5p mimics or the negative control (n = 3). **b.** Flow cytometry analysis and quantification of apoptosis in HepG2 cells transfected with miR-486-5p mimics or the negative control (n = 3). **c.** Quantification of microtubule-associated protein LC3B-II, p62 and Beclin-1 protein levels from the immunoblots shown in Fig. 2e (n = 3). **d.** Quantification of LC3B-II, p62 and Beclin-1 protein levels from the immunoblots shown in Fig. 2f (n = 3). **e.** Quantification of EGFP-positive and mCherry-positive LC3 puncta from the mCherry–EGFP–LC3 reporter images shown in Fig. 2g (n = 6). **f.** Quantification of PTEN protein levels from the immunoblots shown in Fig. 5c (n = 3). **g.** Quantification of p-AKT/AKT, p-mTOR/mTOR, and p-ULK1/ULK1 ratios from the immunoblots shown in Fig. 5e (n = 3). **h.** Immunoblot analysis and quantification of AKT phosphorylation in Hep3B cells following MYB overexpression (n = 3). **i.** Quantification of LC3B-II protein levels from the immunoblots shown in Fig. 5h in cells treated with miR-486-5p mimics with or without AKT or mTOR inhibition (n = 3). Data are presented as the mean ± SD. (*$p < 0.05$; **$p < 0.01$; ***$p < 0.001$; ns, not significant)

Supplementary Figure 2 Additional validation of AKT/mTOR-dependent rescue effects and *in vivo* controls.

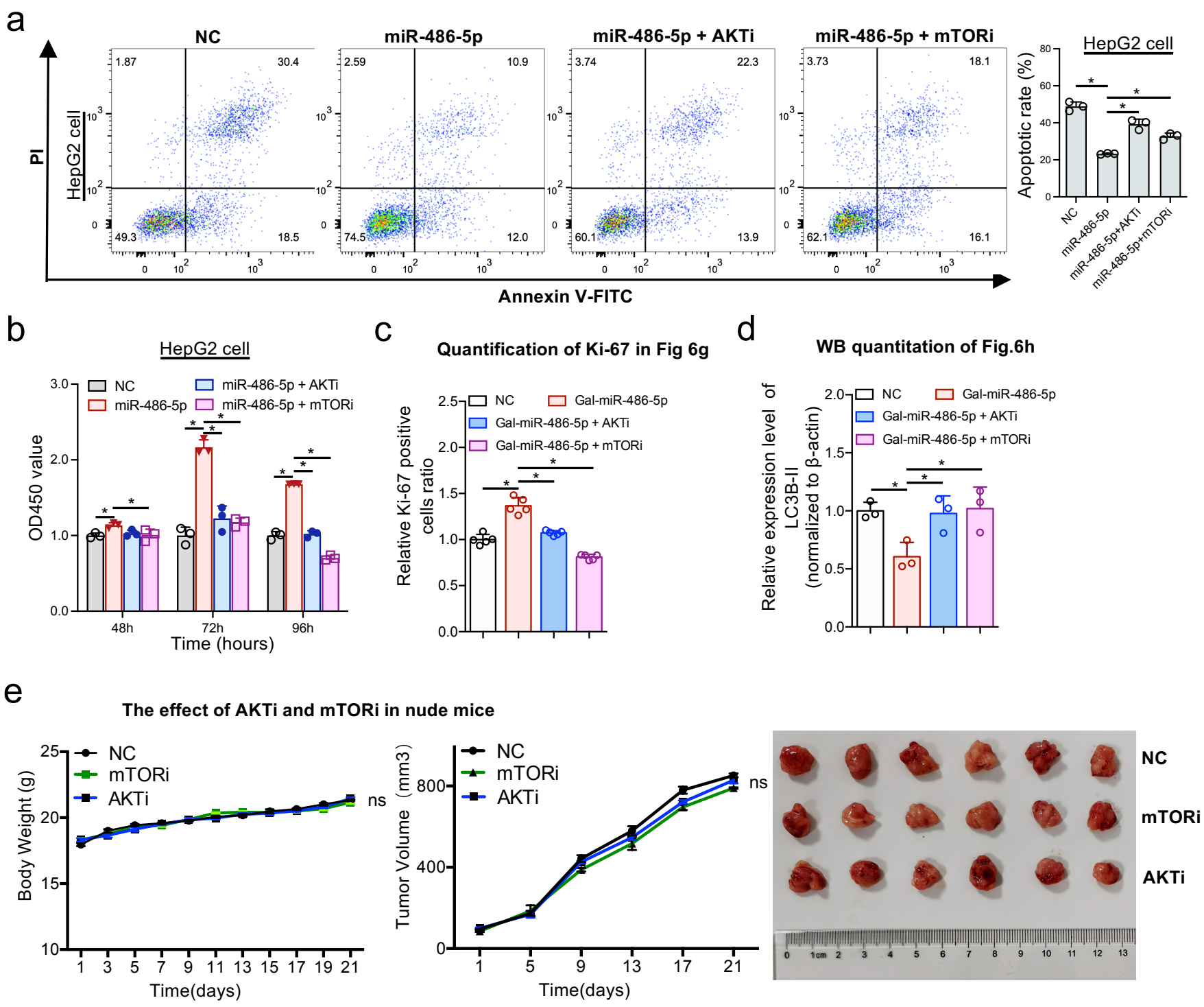

a. Flow cytometry analysis of apoptosis in HepG2 cells following treatment with negative control (NC), miR-486-5p, miR-486-5p+AKT inhibitor (MK-2206), or miR-486-5p+mTOR inhibitor (rapamycin) (n = 3). **b.** CCK-8 assays measuring the viability of HepG2 cells at 48, 72, and 96 h after treatment with NC, miR-486-5p, the miR-486-5p+AKT inhibitor, or the miR-486-5p+mTOR inhibitor (n = 3). **c.** Quantification of Ki-67-positive cells in liver tumour sections from mice treated with NC, Gal-miR-486-5p, Gal-miR-486-5p+AKT inhibitor or Gal-miR-486-5p+mTOR inhibitor (n = 5). **d.** Quantification of LC3B-II protein levels in liver tumour tissues from the indicated treatment groups (n = 3). **e.** Body weight, tumour volume and excised tumours from

nude mice treated with NC, the AKT inhibitor or the mTOR inhibitor (n = 6). The data are presented as the mean ± SD. (**a**, **b**, **c**, **d**). The data are presented as the mean ± SEM. (**e**). (*$p < 0.05$; **$p < 0.01$; ***$p < 0.001$; ns, not significant)

Table Supplement 1. A list of the oligonucleotide sequences for miR-486-5p inhibitor and mimic

Name	Sequence
inhibitor NC	sense: UCUACUCUUUCUAGGAGGUUGUGA<br>antisense: /
hsa-miR-486-5p inhibitor	sense: CUCGGGGCAGCUCAGUACAGGA<br>antisense: /
mimics NC	sense: UCACAACCUCCUAGAAAGAGUAGA<br>antisense: UCUACUCUUUCUAGGAGGUUGUGA
hsa-miR-486-5p mimics	sense: UCCUGUACUGAGCUGCCCCGAG<br>antisense: CUCGGGGCAGCUCAGUACAGGA

Table Supplement 2. A list of the designed sequences for siMYB

Name	Sequence
siMYB-1	sense: ACAGAUGACUGGAAAGUUATT<br>antisense: UAACUUUCCAGUCAUCUGUTT
siMYB-2	sense: CAGAAGAGGAAGACAGAAUTT<br>antisense: AUUCUGUCUUCCUCUUCUGTT

Table Supplement 3. A list of the designed primer sequences for RT-qPCR

Gene	Sequence	
ANK1	Forward	5'-AGGAGGTGACTGTAGAGGGG-3'
	Reverse	5'-GAGGTGTGATCCTTGGAGTGT-3'
miR-486-5p	Forward	5'-CCCCGAGGTCGTATCCAGT-3'
	Reverse	5'-GTATCCAGTGCGTGTCGTGG-3'
MYB	Forward	5'-GAAAGCGTCACTTGGGGAAAA-3'
	Reverse	5'-TGTTCGATTCGGGAGATAATTGG-3'
GAPDH	Forward	5'-CCTGCCTCTACTGGCGCTGC-3'
	Reverse	5'-GCAGTGGGGACACGGA AGGC-3'
U6	Forward	5'-GCTTCGGCAGCACATATACTAAAAT-3'
	Reverse	5'-CGCTTCAGAATTTGCGTGTCAT-3'

Table Supplement 4. A list of the designed primer sequences for ChIP

Gene	Sequence	
ANK1-1	Forward	ACATCTGTGATGTGCAGTCCA
	Reverse	CCTCACAGTCATGCCTCCAT
ANK1-2	Forward	GTTAGAGGCAAGGCCCAGTT
	Reverse	TACCAAGCTGCAGAGAACGA
ANK1-3	Forward	CTTAGGGGAGGCAGAAGGAG
	Reverse	ACGGCTAAGGAGGGAGTTGT
ANK1-4	Forward	TCACTTGATCCTGGATACGTTTT
	Reverse	TTGATTTGCCCCTTTCAGAC
